# Clinical outcome of breast cancer in carriers of *BRCA1* and *BRCA2* mutations according to molecular subtypes

**DOI:** 10.1038/s41598-020-63759-1

**Published:** 2020-04-27

**Authors:** Solene De Talhouet, Julien Peron, Aurelie Vuilleumier, Alex Friedlaender, Valeria Viassolo, Aurélie Ayme, Alexandre Bodmer, Isabelle Treilleux, Noemie Lang, Jean- Christophe Tille, Pierre O. Chappuis, Adrien Buisson, Sophie Giraud, Christine Lasset, Valerie Bonadona, Olivier Trédan, S.Intidhar Labidi-Galy

**Affiliations:** 1 Department of Medical Oncology, Centre Léon Bérard, Lyon, France; 2 Department of Oncology, Hospices Civils de Lyon, Université Lyon 1, Lyon, France; 3 Department of Biostatistics, Hospices Civils de Lyon, CNRS, UMR 5558, Lyon, France; 4 Laboratoire de Biométrie et Biologie Evolutive, Equipe Biostatistique-Santé, F-69100 Villeurbanne, France; 5 Department of Oncology, Hôpitaux Universitaires de Genève, Geneva, Switzerland; 6 Department of Genetic Medicine, Laboratory and Pathology, Hôpitaux Universitaires de Genève, Geneva, Switzerland; 7 Department of Biopathology, Centre Léon Bérard, Lyon, France; 8 Department of Diagnostics, Division of pathology, Hôpitaux Universitaires de Genève, Geneva, Switzerland; 9 Department of Genetics, Hospices Civils de Lyon, Lyon, France; 10 Unit of Prevention and Genetic Epidemiology, UMR CNRS 5558, Centre Léon Bérard, Lyon, France; 11 Department of Medicine, Faculty of Medicine, University of Geneva, Geneva, Switzerland

**Keywords:** Prognostic markers, Breast cancer, Cancer genetics

## Abstract

*BRCA1/BRCA2* genes play a central role in DNA repair and their mutations increase sensitivity to DNA-damaging agents. There are conflicting data regarding the prognostic value of *BRCA* germline mutations in breast cancer (BC) patients. We collected clinical, pathological and genetic data of a cohort 925 BC patients preselected for genetic screening and treated with neoadjuvant or adjuvant chemotherapy, of whom 266 were *BRCA* carriers. Overall, 171 women carried a *BRCA1* mutation, 95 carried a *BRCA2* mutation, and 659 were non-carriers. In the entire cohort, there was a prolonged disease-free survival (DFS) for *BRCA* carriers (hazard ratio (HR) = 0.63; 95% confidence interval (CI), 0.44–0.90 for *BRCA1;* HR = 0.72; 95%CI, 0.47–1.1 for *BRCA2*; *p* = 0.020) and a trend toward prolonged disease-specific survival (DSS; HR = 0.65; 95%CI, 0.40–1.1 for *BRCA1*; HR = 0.78; 95%CI, 0.44–1.38 for *BRCA2*; *p* = 0.19) though not statistically significant. In the TNBC group, *BRCA* carriers had prolonged DFS (adjusted HR = 0.50; 95%CI, 0.28–0.89 *for BRCA1;* adjusted HR = 0.37; 95%CI, 0.11–1.25, *for BRCA2; p* = 0.034) and DSS (adjusted HR = 0.42; 95%CI, 0.21–0.82 for *BRCA1;* adjusted HR = 0.45; 95%CI, 0.11–1.9 for *BRCA2; p* = 0.023). In the non-TNBC group, the *BRCA1* or *BRCA2* mutations did not have any impact on survival. These results suggest that *BRCA1/BRCA2* germline mutations are associated with prolonged survival only if women were diagnosed with TNBC.

## Introduction

*BRCA1/BRCA2* germline mutations account for approximately 5% of all breast cancers^1^. These tumor suppressor genes encode large, ubiquitous and multifunctional proteins that play a central role in DNA repair, cell-cycle control and chromosomal stability^2^. Cells with non-functional BRCA1/BRCA2 proteins are severely impaired in their ability to repair DNA double strand breaks (DSBs) through homologous recombination^2^. As a consequence, tumors harboring deleterious mutations of *BRCA1/BRCA2* genes are highly sensitive to DNA-damaging agents, such as interstrand crosslinking agents (platinum or alkylating agents), topo-isomerase II inhibitors (anthracyclines) or PARP inhibitors^2–4^.

In breast cancer patients, the tumor phenotype differs according to the *BRCA1* or *BRCA2* germline mutation status. *BRCA1* mutation carriers mainly develop triple negative breast cancers (TNBC), whereas *BRCA2* carriers are more likely to develop estrogen receptor (ER) and/or progesterone receptor (PR) positive tumors^5^. Not all *BRCA* carriers who develop breast cancer receive adjuvant chemotherapy, depending on several factors, including tumor stage, grade and molecular subtype. Currently, there are conflicting data regarding the predictive and prognostic values of *BRCA* mutations on the survival of non-metastatic breast cancer patients^6–8^. *BRCA* carriers with TNBC have been shown to be more sensitive to DNA-damaging agents^9–15^ but this did not translate into a survival benefit^6,9,12,16,17^.

*BRCA* germline mutations account for approximately 10–15% of ovarian cancers^18^. The majority of ovarian cancers that develop in *BRCA* carriers (either *BRCA1* or *BRCA2*) are high-grade serous ovarian carcinomas (HGSOC). Ovarian cancers are frequently diagnosed at advanced stages and receive platinum-based chemotherapy^19^. Several studies have shown that among ovarian cancer patients, *BRCA1* and especially *BRCA2* carriers respond better than non-carriers to platinum-based chemotherapy and have prolonged survival^20–22^. We hypothesized that *BRCA* germline mutations would lead to prolonged survival in breast cancer patients treated by DNA-damage agents such as alkylating agents and/or anthracylines^23^. We conducted a multicentric retrospective study with the primary objective of assessing the prognostic value of *BRCA* germline mutation on survival among stage I-III breast cancer patients treated with chemotherapy. Patients were included if they have been selected for genetic testing of *BRCA* germline mutation.

## Results

### Patient demographics and clinical characteristics

From the entire cohort, a total of 925 patients were identified (677 from the French cohort and 248 from the Swiss cohort)(supplementary Figure S1), of whom 659 were non-carriers, 171 were *BRCA1* carriers, and 95 were *BRCA2* carriers (supplementary Table S1). Patient demographics, tumor characteristics, and type of administered chemotherapy are summarized in Table 1. The median age at diagnosis (40 years) was similar between carriers and non-carriers. Most *BRCA1* carriers developed TNBC (68%) compared to 19% among *BRCA2* carriers and 24% among the non-carriers (*p* < 0.0001). *BRCA1* carriers were more likely to develop high grade (*p* < 0.0001) and high mitotic index tumors (*p* < 0.0001). Axillary node involvement was more frequent in *BRCA2* carriers (*p* = 0.016).

**Table 1:** Patients characteristics of the entire cohort.

Variable	All (n = 925)	*BRCA* status			*p*
		Non-carriers (n = 659)	*BRCA1* (n = 171)	*BRCA2* (n = 95)	
Age*, years*, median (25^th^–75^th^) *NA* = 0	40 (34–48)	39 (34–48)	40 (35–49)	40 (35–47)	0.73
cT *(%)*					0.20
cT0	47 (7%)	35 (7%)	8 (6%)	4 (6%)	
cT1	254 (36%)	172 (34%)	59 (42%)	23 (39%)	
cT2	293 (41%)	211 (42%)	53 (38%)	29 (43%)	
cT3	86 (12%)	66 (13%)	13 (9%)	7 (10%)	
cT4 *NA* = 216	29 (4%)	17 (3%)	7 (5%)	5 (7%)	
cN *(%)*					0.20
cN0	477 (68%)	328 (66%)	104 (76%)	44 (66%)	
cN1	210 (30%)	159 (32%)	31 (23%)	20 (29%)	
cN2	8 (1%)	6 (12%)	1 (1%)	1 (1%)	
cN3 *NA* = 222	8 (1%)	6 (12%)	0 (0%)	2 (3%)	
Positive nodes *** *NA* = 22	430 (48%)	312 (48%)	64 (39%)	54 (57%)	0.016
Grade *(%)*					<0.0001
1	45 (5%)	40 (6%)	1 (1%)	4 (4%)	
2	341 (38%)	269 (42%)	33 (20%)	39 (43%)	
3 *NA* = 25	514 (57%)	335 (52%)	132 (80%)	47 (52%)	
Mitotic index *(%)*					<0.0001
1	218 (27%)	182 (31%)	13 (9%)	23 (29%)	
2	247 (30%)	182 (31%)	41 (28%)	24 (31%)	
3 *NA* = 109	351 (43%)	227 (38%)	93 (63%)	31 (40%)	
Positive ER *(%) NA* = 5	554 (60%)	444 (68%)	40 (23%)	70 (76%)	<0.0001
Positive *PR (%) NA* = 5	484 (53%)	386 (59%)	37 (22%)	61 (66%)	<0.0001
Positive HER-2 *(%) NA* = 67	173 (20%)	153 (25%)	7 (5%)	13 (16%)	<0.0001
TNBC (%) NA = 67	270 (31%)	148 (24%)	106 (68%)	16 (19%)	<0.0001
Chemotherapy *(%)*					0.67
Neoadjuvant	254 (27%)	184 (28%)	41 (24%)	29 (31%)	
Adjuvant	662 (72%)	467 (71%)	129 (76%)	66 (69%)	
Both *NA* = 0	9 (1%)	8 (1%)	1 (1%)	0 (0%)	
Anthracyclines *(%) NA* = 4	751 (82%)	529 (80%)	143 (85%)	79 (84%)	0.39
Taxanes *(%) NA* = 4	717 (78%)	526 (80%)	121 (72%)	70 (75%)	0.046
Alkylating agent *(%) NA* = 4	874 (95%)	620 (94%)	162 (96%)	92 (98%)	0.33
Platinum *(%) NA* = 4	31 (3%)	24 (4%)	5 (3%)	2 (2%)	0.87
Trastuzumab *(%) NA* = 4	143 (16%)	131 (20%)	3 (2%)	9 (10%)	<0.0001

*NA*: not available. ER: estrogen receptors, PR: progesterone receptors, HER-2: human epidermal growth factor receptor-2, TNBC: triple negative breast cancers. ***Positive nodes: pN if pre-chemotherapy biopsy positive; yN or nodal scar in the removed lymph node if neoadjuvant chemotherapy.

ER, PR, and HER-2 status were available for 858 patients. Among the 270 who developed TNBC, 106 were *BRCA1* carriers, 16 were *BRCA2* and 148 were non-carriers. Patients and tumor characteristics were comparable between *BRCA* carriers and non-carriers (supplementary Table S2). Among the 588 women who developed non-TNBC, *BRCA1* carriers were older than *BRCA2* and non-carriers (*p* = 0.014; supplementary Table S3). *BRCA1* carriers developed tumors displaying higher grade (*p* = 0.056), and a higher mitotic index (*p* = 0.047) and were less frequently expressing ER (*p* = 0.0053) than *BRCA2* carriers or non-carriers. HER-2 was less frequently overexpressed/amplified in tumors from *BRCA* carriers compared to non-carriers (*p* = 0.004).

### Chemotherapy

The majority of patients received adjuvant chemotherapy (72% for the entire cohort, 66% for TNBC, and 73% for non-TNBC). Most of the patients received two DNA damaging-agents: an alkylating agent (95%) and an anthracycline (82%; Table 1). Non-carriers were more likely to receive taxanes (*p* = 0.046; Table 1), in particular among those who developed TNBC (*p* = 0.0088; supplementary Table S2). Non-carriers more frequently received trastuzumab (*p* < 0.0001; Table 1). Very few patients received platinum derivatives (3%; Table 1).

### Survival estimates

**Figure 1:**
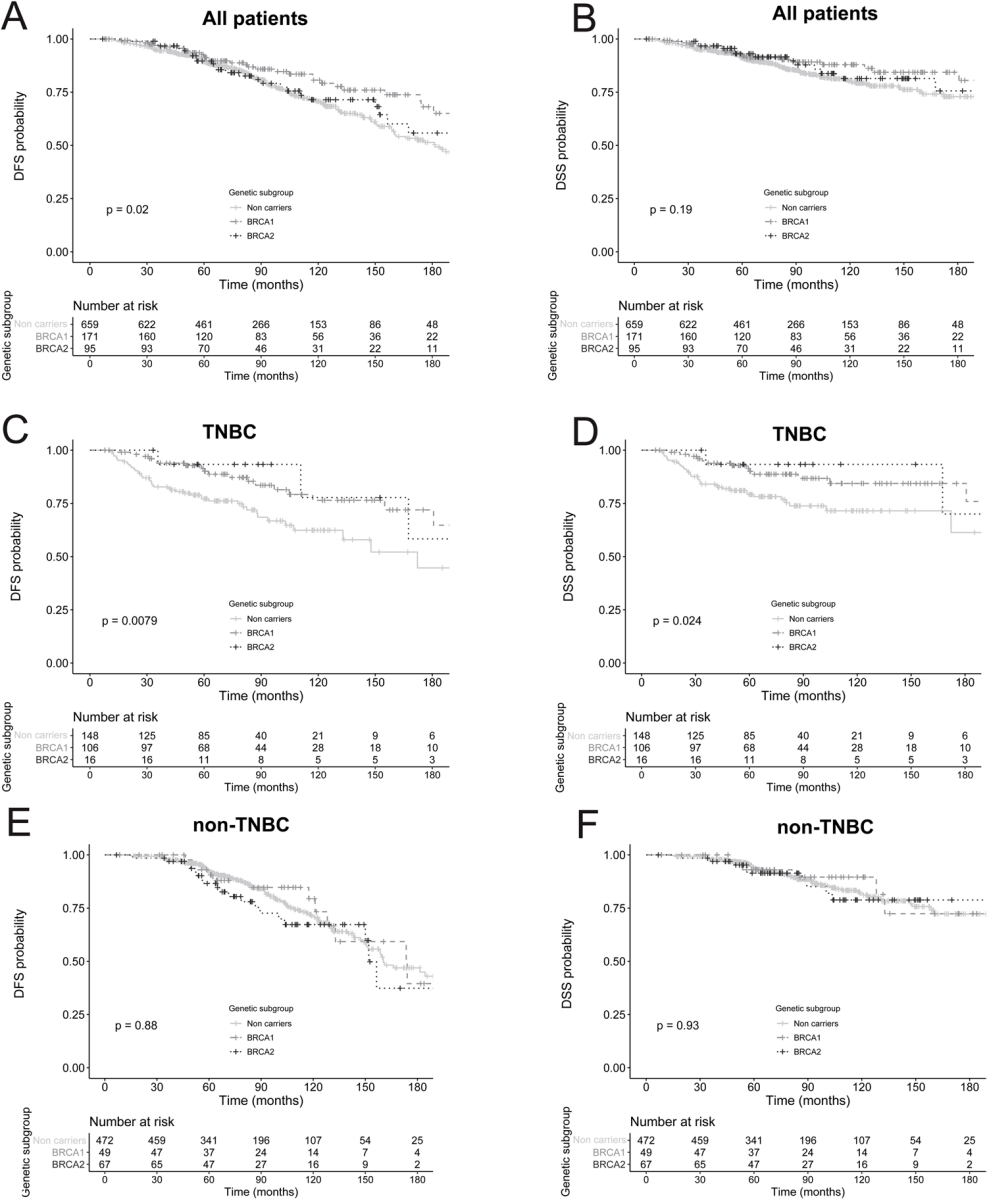
DFS and DSS according to *BRCA1/BRCA2* status and molecular phenotype.

The median follow-up for the entire cohort was 7.3 years (7–7.8). Overall, 237 patients relapsed during the follow-up: 178 non-carriers, 35 *BRCA1*, and 24 *BRCA2* carriers. There were 133 deaths related to breast cancer: 101 non-carriers, 19 *BRCA1* carriers, and 13 *BRCA2* carriers. In the entire cohort (n = 925), there was a prolonged DFS for *BRCA1* (5-year rate 92%; hazard ratio (HR) = 0.63; 95% confidence interval (CI), 0.44–0.90) as well as for *BRCA2* (5-year rate 90%; HR = 0.72; 95%CI, 0.47–1.1; *p* = 0.020; Fig. 1A and Table 2) compared to non-carriers (5-year rate 89%). A trend toward prolonged DSS was observed in *BRCA* carriers (5-year rate 93%; HR = 0.65; 95%CI, 0.40–0.1.1 for *BRCA1*; 5-year rate 93%; HR = 0.78; 95%CI, 0.44–1.38 for *BRCA2*; *p* = 0.19 and a 5-year rate 91% for non-carriers; Fig. 1B and Table 2) though not statistically significant.

**Table 2:** Multivariate analysis of DFS and DSS in the entire cohort.

Cox proportional hazards regression											
	N	Disease-free survival					Disease-specific survival				
			Unadjusted analysis		Adjusted Analysis		5-years DSS rate (95% CI)	Unadjusted analysis		Adjusted Analysis	
		5-years DFS rate (95% CI)	HR (95%CI)	*p*	HR (95%CI)	*p*		HR (95%CI)	*p*	HR (95%CI)	*p*
**BRCA status**											
Non-carriers	659 (71%)	89 (87–92)	1		1		91 (89–37)	1		1	
*BRCA1*	171 (18%)	92 (88–96)	0.63 (0.44–0.90)	0.020	0.63 (0.43–0.92)	0.018	93 (89–97)	0.65 (0.40–1.1)	0.19	0.66 (0.39–1.1)	0.18
*BRCA2*	95 (10%)	90 (83–96)	0.72 (0.47–1.1)		0.70 (0.45–1.1)		93 (88–99)	0.78 (0.44–1.38)		0.74 (0.42–1.3)	
**Grade**											
1	45 (5%)	98 (93–100)	1	0.30	NI	NI	95 (89–100)	1	0.72	NI	NI
2	341 (37%)	89 (86–93)	1.4 (0.76–2.7)				93 (90–96)	1.3 (0.57–3.1)			
3	514 (56%)	89 (86–92)	1.2 (0.64–2.2)				91 (88–93)	1.2 (0.51–2.7)			
**Age**				0.16	NI	NI					
>35	666 (72%)	91 (88–93)	1				92 (90–94)	1	0.26	NI	NI
≤35	258 (28%)	88 (84–92)	1.2 (0.93–1.6)				98 (86–94)	1.2 (0.86–1.8)			
**Nodal status**											
Negative	473 (52%)	93 (90–95)	1	0.0029	1	0.0032	95 (92–97)	1	<0.0001	1	<0.0001
Positive	430 (48%)	87 (84–91)	1.5 (1.1–1.9)		1.5 (1.1–1.9)		89 (86–92)	2.1 (1.5–3.1)		2.1 (1.4–3.0)	

NI: not-included.

Subgroup analysis by molecular subtype revealed that *BRCA* carriers had significantly prolonged DFS and DSS in the TNBC subgroup only (n = 270; Table 3). After adjustment for nodal status, *BRCA1* (5-year rate 91%; HR = 0.50; 95%CI, 0.28–0.89) and *BRCA2* carriers (5-year rate 93%; HR = 0.37; 95%CI, 0.11–1.25) had prolonged DFS compared to non-carriers (5-year rate 77%; *p* = 0.034; Table 3 and Fig. 1C). *BRCA1* (5-year rate 91%; HR = 0.42; 95%CI, 0.21–0.82) and *BRCA2* carriers (5-year rate 93%; HR = 0.45; 95%CI, 0.11–1.9) consistently had prolonged DSS compared to non-carriers (5-year rate 79%; *p* = 0.023; Table 3 and Fig. 1D). The landmark analysis at one year performed as a sensitivity analysis was consistent with this estimated impact of BRCA status on DFS and DSS in the TNBC” (supplementary Table 4). For women with non-TNBC, the *BRCA1* (5-year rate 91%; HR = 0.91; 95%CI, 0.50–1.7) or *BRCA2* (5-year rate 87%; HR = 1.1; 95%CI, 0.70–1.9) status did not have any impact on DFS (*p* = 0.88; supplementary Table S5 and Fig. 1E). Similarly, the *BRCA1/BRCA2* status did not have any impact on the 5-year DSS (*p* = 0.93; supplementary Table S5 and Fig. 1F) in the multivariate analysis.

**Table 3:** Multivariate analysis of DFS and DSS in TNBC.

Cox proportional hazards regression											
	N	Disease-free survival					Disease-specific survival				
		5-years DFS rate (95% CI)	Unadjusted analysis		Adjusted Analysis			Unadjusted analysis		Adjusted Analysis	
			HR (95%CI)	*p*	HR (95%CI)	*p*	5-years DSS rate (95% CI)	HR (95%CI)	*p*	HR (95%CI)	*p*
**BRCA status**											
Non-carriers	148 (55%)	77 (70–84)	1		1		79 (73–86)	1		1	0.023
*BRCA1*	106 (39%)	91 (86–97)	0.47 (0.28–0.81)	0.0079	0.50 (0.28–0.89)	0.034	91 (86–97)	0.45 (0.24–0.85)	0.024	0.42 (0.21–0.82)	
*BRCA2*	16 (6%)	93 (82–100)	0.34 (0.10–1.1)		0.37 (0.11–1.25)		93 (82–100)	0.39 (0.09–1.6)		0.45 (0.11–1.9)	
**Grade**											
1	3 (1%)	100 (100–100)	1		1	0.0023	100 (100–100)	1	0.028	NI*	NI
2	44 (16%)	65 (51–82)	0.69 (0.09–5.2)		0.0 (0.0-NA)		69 (56–86)	0.79 (0.10–6.1)			
3	220 (82%)	87 (82–92)	0.49 (0.28–0.85)	0.040	0.40 (0.22–0.72)		87 (83–92)	0.44 (0.24–0.82)			
**Age**											
>35	202 (75%)	84 (78–89)	1	0.81	NI	NI	85 (80–90)	1	0.61	NI	NI
≤35	66 (25%)	84 (75–94)	0.93 (0.53–1.64)				84 (75–94)	0.83 (0.43–1.6)			
**Nodal status**											
Negative	186 (72%)	90 (86–95)	1	0.00010	1	<0.0001	91 (87–96)	1	<0.0001	1	<0.0001
Positive	73 (28%)	69 (59–81)	2.7 (1.6–4.4)		3.1 (1.9–5.1)		71 (60–82)	3.4 (1.9–6.0)		3.3 (1.9–6.0)	

NI: not included.

### Response to neoadjuvant chemotherapy

Of the 263 (28%) patients who received neoadjuvant chemotherapy, the ER, PR and HER-2 status was available in 250 patients (95%). The pCR rate was significantly higher in *BRCA1* (45%) compared to *BRCA2* carriers (28%) and non-carriers (25%; *p* = 0.040; Table 4). Subgroup analysis by molecular subtype revealed that *BRCA1* (54%) and *BRCA2* carriers (57%) had significantly increased chemosensitivity compared to non-carriers (25%; *p* = 0.015) in the TNBC-subgroup only. In the HER-2 positive and the ER/PR positive/HER-2 negative subgroups, there was no difference between *BRCA1/BRCA2* carriers and non-carriers regarding the pCR rate.

**Table 4:** Pathologic complete response according to *BRCA* status and molecular subtype.

	pCR rate			
	Non-carriers N (%)	*BRCA1* N (%)	*BRCA2* N (%)	*p*
Entire cohort	48/192 (25%)	18/40 (45%)	8/29 (28%)	0.040
TNBC	13/53 (25%)	15/28 (54%)	4/7 (57%)	0.015
HER-2 positive *	24/68 (35%)	1/3 (33%)	2/6 (33%)	1.0
ER/PR positive, HER-2 negative	9/62 (15%)	1/6 (17%)	1/14 (7%)	0.74

pCR: pathologic complete response. *HER-2 status missing in 12 cases.

## Discussion

In the current study, we observed better disease-free survival of breast cancer patients who were selected for genetic screening, treated by chemotherapy and are *BRCA* carriers. Subgroup analysis revealed that the *BRCA* germline mutation is an independent prognostic factor associated with prolonged survival (both DFS and DSS) only for women with TNBC. For those who had ER/PR positive and/or HER-2 positive tumors (non-TNBC), *BRCA* mutations did not have any impact on outcome.

TNBC, mostly belonging to the basal-like subtype, share several molecular features of HGSOC including high levels of genomic instability and frequent *TP53* mutations^19,24,25^. The majority of HGSOC patients are diagnosed at advanced stages and receive platinum-based chemotherapy^19^. *BRCA* carriers who developed HGSOC have increased survival compared to non-carriers^19–21^. This survival benefit has been linked to impaired DNA DSBs repair and consequently increased sensitivity to platinum^26^. For breast cancer patients, there are conflicting results regarding the prognosis and the predictive value of the *BRCA* germline status due to several issues: i) the phenotype of the tumor varies whether it is a *BRCA1* (mainly TNBC) or a *BRCA2* (mainly ER/PR positive) mutations; ii) adjuvant chemotherapy is not systematic and depends, among other characteristics, on tumor stage, grade, and molecular subtypes. Overall, it seems that *BRCA1* carriers have poorer survival, probably due to the fact that they frequently develop TNBC, whereas *BRCA2* germline mutation was not found to have a prognostic impact^8,27^. Whereas the prognostic value depends on tumor characteristics, the predictive value depends on the administered treatment.

DNA interstrand crosslinks (ICLs) are among the most lethal lesions to DNA. They are generated by several chemotherapeutic drugs such as platinum, mitomycine and alkylating agents. Although these drugs are backbone therapy of multiple cancers, it is well after their introduction to the clinics that it was discovered that they act by inducing ICLs^28^. Cells defective in *BRCA* genes are highly sensitive to drugs that generate ICLs such as bifunctional alkylating agents and platinum^28–30^. Another chemotherapeutic drugs that have biological background for efficacy in *BRCA* mutated tumors are topo-isomerase II inhibitors like anthracyclines^31,32^. Sensitivity to anthracylines and alkylating agents in *BRCA* carriers with breast cancer are emphasized by recent reports from INFORM and GeparOcto clinical trials^15,23^.

We hypothesized that among breast cancer patients who received DNA damage agents *BRCA* carriers will be more chemosensitive and this could translate into survival benefit. A quarter of the patients in our cohort received neoadjuvant chemotherapy. We observed that pCR rates significantly differ according to *BRCA1/BRCA2* status and molecular subtype. For TNBC, our data are consistent with previous reports showing increased pCR rate in *BRCA1*^9–12,33^ and/or *BRCA2* carriers^10,33,34^. However, less than half of *BRCA* carriers would develop TNBC^35^, 45% in our cohort, and few are known on chemosensitivity of *BRCA2* carriers. We did not observe any impact of *BRCA* mutations on pCR in HER-2 positive or ER/PR-positive HER-2 negative tumors. ER/PR-positive tumors in *BRCA2* carriers seemed resistant to chemotherapy with a response rate estimated to 7% only. Our observations should be interpreted cautiously given the limited number of patients in each subgroup and the highly selected population. Nevertheless, it suggests that chemosensitivity in *BRCA* carriers may dramatically vary with the molecular phenotype of the tumor^10,34,36^.

We observed a survival benefit in *BRCA1/BRCA2* carriers who developed TNBC. There are contradicting results regarding the survival benefit of *BRCA* mutations in TNBC^6,9,12,16,17,33^. Plausible explanations are: i) we did not exclude *BRCA2* carriers and they are rare compared to *BRCA1*, ii) our cohort of *BRCA1/BRCA2* carriers who developed TNBC included more than 100 *BRCA* carriers. We did not observe any survival benefit in *BRCA1/BRCA2* carriers with HER-2 positive or ER/PR-positive HER-2 negative breast cancers (non-TNBC). This result was unexpected and mirrors the response rates to neoadjuvant chemotherapy in the different subgroups. It suggests the existence of different types of breast tumors arising in *BRCA* carriers with distinct responses to DNA-damaging agents. Investigating the molecular mechanisms underlying these differences, such as mutational signatures^37^, somatic loss of the wild-type allele^38^, *BRCA* genotype, the references doi: 10.1007/s10549-018-05127-2 and doi: 10.1158/1078-0432 recombination deficiency scores and/or infiltration by lymphocytes^39,40^ are important questions that need to be addressed in the future.

Our results are consistent with the recently published POSH study, a large prospective cohort (>2,700) that addressed the prognostic value of *BRCA* mutations in young women (<40 years). The majority of participants (89%) and virtually all cases of TNBC (98%) received chemotherapy. The POSH study showed survival benefit only in *BRCA* carriers who had developed TNBC and this benefit was observed in the first two years following diagnosis^35^. The POSH study brings new insights into the prognostic value of *BRCA* mutations in the context of breast cancer in young women treated by chemotherapy. The Geparquinto trial consistently showed survival benefit from *BRCA* germline mutations in TNBC^33^.

Our study had several limitations. It is a retrospective study that included patients screened for *BRCA1/BRCA2* germline mutations. We recruited only women who were preselected based on their personal or family history that suggests a genetic predisposition. There might be a very specific additional risk factor profile for both environmental and genetic factors in these patients^41,42^. In the French cohort we included all *BRCA* carriers and a subgroup of non-carriers who were randomly selected. This lead to a substantial enrichment of *BRCA* carriers among women with TNBC (45%), much higher than expected for unselected TNBC^10,33^. These biases are reflected by the young age of our cohort that does not represent the general population of breast cancer patients. There is a survival bias related to the time from cancer diagnosis to genetic testing. We excluded women who did not receive adjuvant chemotherapy and thus could not address the prognostic value of the *BRCA* status among this population. We probably missed a substantial proportion of *BRCA* carriers who did not undergo genetic screening due to the absence of personal or family history^35,37^. Moreover, this study does not include a central review of pathology data. Nevertheless, *BRCA* carriers in our cohort had clinical and pathological characteristics consistent with previous reports^5,7^.

The strengths of our study are the following: we conducted a multicentric, international study with patients recruited in cancer comprehensive center, university hospitals, and private clinics. All patients underwent complete *BRCA1* and *BRCA2* gene sequencing, avoiding a selection bias in studies with founder mutations only^6^. We analyzed separately the impact of *BRCA1* and *BRCA2* mutations on survival and pCR and we did not focus on one molecular subtype or chemotherapy regimen or setting.

In summary, our study suggests that the prognostic value of *BRCA1/BRCA2* germline mutations in breast cancer patients who were preselected for genetic screening and treated with neoadjuvant or adjuvant chemotherapy depends on the molecular subtype with a survival benefit only in women with TNBC.

## Methods

### Patient population

Women with non-metastatic invasive breast cancers who had been preselected for genetic screening for *BRCA1/BRCA2* germline mutation and who received neoadjuvant or adjuvant chemotherapy were included in this study. *BRCA* status was determined at the Centre Léon Bérard, the Hospices Civils de Lyon, Lyon, France (1995–2014; French cohort) and the Hôpitaux Universitaires de Genève (1995–2016; Swiss cohort). From Geneva, all women (*BRCA* carriers and non-carriers) who met the inclusion criteria were included. In order to reduce the number of non-*BRCA* carriers in the study cohort, all *BRCA* carriers and a subgroup of non-carriers diagnosed in Lyon (randomly selected) were included. A protocol with a standardized case report form was used for all data collection and submitted to the Geneva Commission cantonale d’ethique de la recherche (CCER 15–158). The study protocol was approved by the Geneva Commission cantonale d’ethique de la recherche and the local institutional review boards in both hospitals in France. Informed written consent was obtained from all patients in the French cohort, and all living patients in the Swiss cohort. The research was performed in accordance with relevant guidelines/regulations. Exclusion criteria were the absence of neoadjuvant or adjuvant chemotherapy, no genetic screening, no follow-up or metastatic disease at diagnosis.

### Data collection

Patient and treatment characteristics were collected from the medical records of patients treated at the Centre Leon Bérard, the Hospices Civils de Lyon, the Hôpitaux Universitaires de Genève and among 7 medical oncologists in private clinics in Geneva, Switzerland. We recorded date of birth, date of diagnosis, chemotherapy regimen, and timing (neoadjuvant or adjuvant). Chemotherapy agents were classified as anthracyclines, alkylating agents, taxanes, or platinum. Trastuzumab and hormonal therapy administration was recorded.

Tumor characteristics were collected from pathological reports. This included histological subtype, grade, estrogen and progesterone receptors status (positivity was defined as nuclear staining of >1% by immunohistochemistry (IHC)), HER-2 status (defined as either 3+ by IHC or as assessed by gene amplification through fluorescence or chromogenic *in situ* hybridization). TNBC were defined as ER, PR and HER-2 negative tumors. Non-TNBC were defined as ER/PR and/or HER-2 positive tumors.

TNM staging was evaluated according to the timing of chemotherapy. If the patient received adjuvant chemotherapy, the pTNM was recorded. If the patient received neoadjuvant chemotherapy, the cTNM and yTNM were recorded. Axillary lymph nodes were considered positive if a pre-chemotherapy biopsy was positive or if there was at least one yN+ or the presence of a histological scar in the removed lymph nodes after neoadjuvant chemotherapy.

### Genetic analysis

Women were referred to the genetic unit for complete *BRCA1* and *BRCA2* germline screening based on the presence of personal history of breast cancer presented at a young age, or the display of a particular tumor phenotype (TNBC) or association with ovarian cancer, or in the context of a positive family history. Blood samples for germline DNA testing were obtained after a signed consent. All participants were screened for *BRCA1* and *BRCA2* mutations. *BRCA1* and *BRCA2* variants were classified as pathogenic according to the ENIGMA *BRCA1/2* Gene Variant Classification Criteria (http://www.enigmaconsortium.org/). Women with variants of uncertain significance were considered as non-carriers.

### Outcome measures

The primary objectives were to compare Disease-free survival (DFS) and Disease-specific survival (DSS) among breast cancer patients according to *BRCA* germline mutations. Secondary objectives were to compare i) DSS and DFS according to *BRCA* status in the TNBC and the non-TNBC population; ii) pCR according to molecular subtype (TNBC vs non-TNBC) and *BRCA* status in the subgroup of patients who received neoadjuvant chemotherapy.

### Statistical analyses

Based on a sample size of 600 non-carriers, 150 *BRCA1* carriers and 100 *BRCA2* carriers, a 80% 5-year DFS among non-carriers and a median follow-up of 6 years, the study had a 93% power to show an improvement of the 5-year DFS from 80% among non-carriers to 89% among the *BRCA* carriers (translating in a hazard ratio of 0.5) at a 2-sided alpha risk of 5%.

DFS was calculated from the time of diagnosis until the date of first documented local, regional, or distal invasive recurrence or death from breast cancer, or to the time of last follow-up. DSS was defined as the time from diagnosis to death caused by breast cancer. Survival outcomes were estimated using the Kaplan–Meier product-limit method and compared by a long-rank test. Cox proportional-hazards (PH) models were fitted to determine the association of the *BRCA* germline status (with time to event outcomes before and after adjustment for significant patient and clinical characteristics. The proportional hazards hypothesis was assessed both graphically and statistically. Cox proportional-hazards models were used for all analyses given the absence of significant deviation from the PH hypothesis in all subgroups and for all reported outcome measures. The following prognostic variables were assessed in univariate analyses: *BRCA* status, age (≤ or> 35 years of age), lymph node status, SBR grade. Variables yielding *p* values less than 0.1 by univariate analysis were retained for the multivariate analysis. The proportional hazards assumption was assessed using scaled Schoenfeld residuals. Because of the high correlation between grade and lymph node involvement and in order to avoid colinearity, grade was not included in the multivariate model. *P* values of ≤ 0.05 were considered statistically significant. As a sensitivity analysis of the main outcomes, a landmark analysis was conducted to exclude patients with DFS or DSS of less than 12 months, in order to avoid any immortal time bias related to the time between the cancer diagnosis and the time of the genetic counseling/testing.

Pathological complete response (pCR) was defined as the absence of any invasive disease in the breast and in the ipsilateral axillary lymph nodes (ypT0/is ypN0) in accordance with the Union for International Cancer Control TNM system^43^. Patient or tumor characteristics and chemotherapy regimens were compared according to the *BRCA* germline status using *χ*^2^ tests or the Fisher’s exact test for categorical variables, and non-parametric Kruskall-Wallis tests for continuous variables. All statistical analyses were carried out using the R software version 3.3.1 (http://www.r-project.org/).

## Supplementary information

Supplementary Tables S1-S5 and Supplementary Figure S1.

## Data Availability

All data analyzed during the study has been included in the manuscript (and its supplementary information files).

## References

[1] Rebbeck TR (2015). Association of type and location of BRCA1 and BRCA2 mutations with risk of breast and ovarian cancer. JAMA.

[2] Roy R, Chun J, Powell SN (2012). BRCA1 and BRCA2: different roles in a common pathway of genome protection. Nat. Rev. Cancer.

[3] Farmer H (2005). Targeting the DNA repair defect in BRCA mutant cells as a therapeutic strategy. Nature.

[4] Robson M (2017). Olaparib for Metastatic Breast Cancer in Patients with a Germline BRCA Mutation. N Engl J Med.

[5] Mavaddat N (2012). Pathology of breast and ovarian cancers among BRCA1 and BRCA2 mutation carriers: results from the Consortium of Investigators of Modifiers of BRCA1/2 (CIMBA). Cancer epidemiology, biomarkers & prevention: a publication of the American Association for Cancer Research, cosponsored by the American Society of Preventive Oncology.

[6] Rennert G (2007). Clinical outcomes of breast cancer in carriers of BRCA1 and BRCA2 mutations. N Engl J Med.

[7] Goodwin PJ (2012). Breast cancer prognosis in BRCA1 and BRCA2 mutation carriers: an International Prospective Breast Cancer Family Registry population-based cohort study. Journal of clinical oncology: official journal of the American Society of Clinical Oncology.

[8] Zhong Q, Peng HL, Zhao X, Zhang L, Hwang WT (2015). Effects of BRCA1- and BRCA2-related mutations on ovarian and breast cancer survival: a meta-analysis. Clinical cancer research: an official journal of the American Association for Cancer Research.

[9] Tung N (2014). Outcome of triple negative breast cancer: comparison of sporadic and BRCA1-associated cancers. Breast Cancer Res Treat.

[10] Hahnen E (2017). Germline Mutation Status, Pathological Complete Response, and Disease-Free Survival in Triple-Negative Breast Cancer: Secondary Analysis of the GeparSixto Randomized Clinical Trial. JAMA Oncol.

[11] Chappuis PO (2002). A significant response to neoadjuvant chemotherapy in BRCA1/2 related breast cancer. Journal of medical genetics.

[12] Wang C (2015). Prevalence of BRCA1 mutations and responses to neoadjuvant chemotherapy among BRCA1 carriers and non-carriers with triple-negative breast cancer. Annals of oncology: official journal of the European Society for Medical Oncology.

[13] Isakoff SJ (2015). TBCRC009: A Multicenter Phase II Clinical Trial of Platinum Monotherapy With Biomarker Assessment in Metastatic Triple-Negative Breast Cancer. Journal of clinical oncology: official journal of the American Society of Clinical Oncology.

[14] Telli ML (2015). Phase II Study of Gemcitabine, Carboplatin, and Iniparib As Neoadjuvant Therapy for Triple-Negative and BRCA1/2 Mutation-Associated Breast Cancer With Assessment of a Tumor-Based Measure of Genomic Instability: PrECOG 0105. Journal of clinical oncology: official journal of the American Society of Clinical Oncology.

[15] Pohl-Rescigno, E. *et al*. Association of Germline Variant Status With Therapy Response in High-risk Early-Stage Breast Cancer: A Secondary Analysis of the GeparOcto Randomized Clinical Trial. JAMA Oncol, 10.1001/jamaoncol.2020.0007 (2020). 10.1001/jamaoncol.2020.0007 PMC7068666 32163106

[16] Bayraktar S (2011). Outcome of triple-negative breast cancer in patients with or without deleterious BRCA mutations. Breast cancer research and treatment.

[17] Paluch-Shimon S (2016). Neo-adjuvant doxorubicin and cyclophosphamide followed by paclitaxel in triple-negative breast cancer among BRCA1 mutation carriers and non-carriers. Breast cancer research and treatment.

[18] Alsop K (2012). BRCA mutation frequency and patterns of treatment response in BRCA mutation-positive women with ovarian cancer: a report from the Australian Ovarian Cancer Study Group. J. Clin. Oncol..

[19] Cancer Genome Atlas Research N (2011). Integrated genomic analyses of ovarian carcinoma. Nature.

[20] Yang D (2011). Association of BRCA1 and BRCA2 mutations with survival, chemotherapy sensitivity, and gene mutator phenotype in patients with ovarian cancer. JAMA.

[21] Bolton KL (2012). Association between BRCA1 and BRCA2 mutations and survival in women with invasive epithelial ovarian cancer. JAMA.

[22] Norquist BM (2016). Inherited Mutations in Women With Ovarian Carcinoma. JAMA oncology.

[23] Tung, N. *et al*. TBCRC 031: Randomized Phase II Study of Neoadjuvant Cisplatin Versus Doxorubicin-Cyclophosphamide in Germline BRCA Carriers With HER2-Negative Breast Cancer (the INFORM trial). *J. Clin. Oncol.*, JCO1903292, 10.1200/JCO.19.03292 (2020). 10.1200/JCO.19.03292 PMC8462533 32097092

[24] Cancer Genome Atlas N (2012). Comprehensive molecular portraits of human breast tumours. Nature.

[25] Manie E (2009). High frequency of TP53 mutation in BRCA1 and sporadic basal-like carcinomas but not in BRCA1 luminal breast tumors. Cancer Res.

[26] Lord, C. J. & Ashworth, A. BRCAness revisited. *Nat. Rev. Cancer* 16, 110–120. 10.1038/nrc.2015.21 26775620

[27] Baretta Z, Mocellin S, Goldin E, Olopade OI, Huo D (2016). Effect of BRCA germline mutations on breast cancer prognosis: A systematic review and meta-analysis. Medicine.

[28] Deans AJ, West SC (2011). DNA interstrand crosslink repair and cancer. Nat. Rev. Cancer.

[29] Turner N, Tutt A, Ashworth A (2005). Targeting the DNA repair defect of BRCA tumours. Curr Opin Pharmacol.

[30] Tutt AN (2005). Exploiting the DNA repair defect in BRCA mutant cells in the design of new therapeutic strategies for cancer. Cold Spring Harb Symp Quant Biol.

[31] Tan, D. S. & Kaye, S. B. Chemotherapy for Patients with BRCA1 and BRCA2-Mutated Ovarian Cancer: Same or Different? *Am Soc Clin Oncol Educ Book*, 114–121, 10.14694/EdBook_AM.2015.35.114 (2015). 10.14694/EdBook_AM.2015.35.114 25993149

[32] Kaye SB (2012). Phase II, open-label, randomized, multicenter study comparing the efficacy and safety of olaparib, a poly (ADP-ribose) polymerase inhibitor, and pegylated liposomal doxorubicin in patients with BRCA1 or BRCA2 mutations and recurrent ovarian cancer. J. Clin. Oncol..

[33] Fasching PA (2018). BRCA1/2 Mutations and Bevacizumab in the Neoadjuvant Treatment of Breast Cancer: Response and Prognosis Results in Patients With Triple-Negative Breast Cancer From the GeparQuinto Study. J. Clin. Oncol..

[34] Bignon, L. *et al*. Efficacy of anthracycline/taxane-based neo-adjuvant chemotherapy on triple-negative breast cancer in BRCA1/BRCA2 mutation carriers. *The breast journal*, 10.1111/tbj.12887 (2017). 10.1111/tbj.12887 28929593

[35] Copson ER (2018). Germline BRCA mutation and outcome in young-onset breast cancer (POSH): a prospective cohort study. Lancet Oncol.

[36] Arun B (2011). Response to neoadjuvant systemic therapy for breast cancer in BRCA mutation carriers and noncarriers: a single-institution experience. J. Clin. Oncol..

[37] Davies H (2017). HRDetect is a predictor of BRCA1 and BRCA2 deficiency based on mutational signatures. Nat Med.

[38] Maxwell KN (2017). BRCA locus-specific loss of heterozygosity in germline BRCA1 and BRCA2 carriers. Nat Commun.

[39] Smid M (2016). Breast cancer genome and transcriptome integration implicates specific mutational signatures with immune cell infiltration. Nat Commun.

[40] Lakhani SR (1998). Multifactorial analysis of differences between sporadic breast cancers and cancers involving BRCA1 and BRCA2 mutations. J Natl Cancer Inst.

[41] Couch FJ (2013). Genome-wide association study in BRCA1 mutation carriers identifies novel loci associated with breast and ovarian cancer risk. PLoS Genet.

[42] Fasching PA (2012). The role of genetic breast cancer susceptibility variants as prognostic factors. Hum Mol Genet.

[43] Control, T. U. f. I. C. AJCC cancer staging manual (Springer, 2010).

