# Publisher Correction: Clinical outcome of breast cancer in carriers of *BRCA1* and *BRCA2* mutations according to molecular subtypes

**DOI:** 10.1038/s41598-020-76385-8

**Published:** 2020-11-02

**Authors:** Solene De Talhouet, Julien Peron, Aurelie Vuilleumier, Alex Friedlaender, Valeria Viassolo, Aurélie Ayme, Alexandre Bodmer, Isabelle Treilleux, Noemie Lang, Jean-Christophe Tille, Pierre O. Chappuis, Adrien Buisson, Sophie Giraud, Christine Lasset, Valerie Bonadona, Olivier Trédan, S. Intidhar Labidi-Galy

**Affiliations:** 1 Department of Medical Oncology, Centre Léon Bérard, Lyon, France; 2 Department of Oncology, Hospices Civils de Lyon, Université Lyon 1, Lyon, France; 3 Department of Biostatistics, Hospices Civils de Lyon, CNRSUMR 5558, Lyon, France; 4 Laboratoire de Biométrie Et Biologie Evolutive, Equipe Biostatistique-Santé, 69100 Villeurbanne, France; 5 Department of Oncology, Hôpitaux Universitaires de Genève, Geneva, Switzerland; 6 Department of Genetic Medicine, Laboratory and Pathology, Hôpitaux Universitaires de Genève, Geneva, Switzerland; 7 Department of Biopathology, Centre Léon Bérard, Lyon, France; 8 Department of Diagnostics, Division of Pathology, Hôpitaux Universitaires de Genève, Geneva, Switzerland; 9 Department of Genetics, Hospices Civils de Lyon, Lyon, France; 10 Unit of Prevention and Genetic Epidemiology, UMR CNRS 5558, Centre Léon Bérard, Lyon, France; 11 Department of Medicine, Faculty of Medicine, University of Geneva, Geneva, Switzerland

Correction to: *Scientific Reports* 10.1038/s41598-020-63759-1, published online 27 April 2020

In the original version of this Article, the author Solene De Talhouet was incorrectly indexed. This error has now been corrected.

Additionally, this Article contained errors in the Reference list. References 41–45 were incorrectly listed as References 39–43.

References 39 and 40 were omitted and are listed below:

Friedlaender, A., *et al., BRCA1/BRCA2* germline mutations and chemotherapy-related hematological toxicity in breast cancer patients. *Breast Cancer Res Treat* **174**, 775–783 (2019).

Labidi-Galy, S.I., *et al.,* Location of mutation in *BRCA2* gene and survival in patients with ovarian cancer. *Clin Cancer Res* **24**, 326–333 (2018).

Furthermore, the Discussion section contained a typographical error.

“Investigating the molecular mechanisms underlying these differences, such as mutational signatures^37^, somatic loss of the wild-type allele^38^, *BRCA* genotype, the references https://doi.org/10.1007/s10549-018-05127-2 and https://doi.org/10.1158/1078-0432 recombination deficiency scores and/or infiltration by lymphocytes^39,40^ are important questions that need to be addressed in the future.”

now reads:

“Investigating the molecular mechanisms underlying these differences, such as mutational signatures^37^, somatic loss of the wild-type allele^38^, *BRCA* genotype^39,40^, homologous recombination deficiency scores and/or infiltration by lymphocytes^41,42^ are important questions that need to be addressed in the future.”

These errors have now been corrected in the HTML and PDF versions of the Article.

