# Supplementary material for: Clinical outcome of breast cancer in carriers of BRCA1 and BRCA2 mutations according to molecular subtypes
Source: Sci Rep. 2020 Apr 27;10:7073. doi: 10.1038/s41598-020-63759-1 (PMC7184602; doi:10.1038/s41598-020-63759-1)
Supplement: Supplementary file 1 — Supplementary Tables S1-S5 and Supplementary Figure S1. [file 41598_2020_63759_MOESM1_ESM.pdf]

Clinical outcome of breast cancer in carriers of *BRCA1* and *BRCA2* mutations according to molecular subtypes

Solene De Talhouet[1], Julien Peron[2,3,4], Aurelie Vuilleumier[5], Alex Friedlaender[5], Valeria Viassolo[5], Aurélie Ayme[6], Alexandre Bodmer[5], Isabelle Treilleux[7], Noemie Lang[5], Jean-Christophe Tille[8], Pierre O. Chappuis[5,6], Adrien Buisson[7], Sophie Giraud[9], Christine Lasset[10], Valerie Bonadona[10], Olivier Tredan[1], S. Intidhar Labidi-Galy[5, 11]

Supplementary materials

Supplementary Table S1: List of BRCA1 and BRCA2 mutations

Supplementary Table S2: Patients characteristics in the triple-negative breast cancers (TNBC) cohort. *NA*: not-available. *** Positive nodes if pre-chemotherapy biopsy positive or there was at least one yN or scar in the removed node after neoadjuvant chemotherapy.

Supplementary Table S3: Patients characteristics in the non-TNBC cohort.

Supplementary Table S4: Landmark analysis at one year of the impact of *BRCA* status on DFS and DSS in the TNBC cohort

Supplementary Table S5: Multivariate analysis of DFS and DSS in the subgroup of patients with non-TNBC. NI: not-included.

Supplementary Figure S1: Flow diagram for selection of patients in the Swiss and French cohorts.

Supplementary Table S1: List of BRCA1 and BRCA2 mutations

BRCA gene	cDNA	protein
1	c.2612_2613delinsTTT	p.(Pro871Leufs*32)
1	c.3143del	p.(Gly1048Valfs*14)
1	c.5266dup	p.(Gln1756Profs*74)
1	c.5106del	p.(Lys1702Asnfs*4)
1	c.4065_4068del	p.(Asn1355Lysfs*10)
1	c.1660G>T	p.(Glu554*)
1	c.220C>T	p.(Gln74*)
1	c.(80+1_81-1)_(4986+1_4987-1)del	p.?
1	c.5128G>T	p.(Gly1710*)
1	c.2211dup	p.(Val738Serfs*2)
1	c.3477_3480del	p.(Ile1159Metfs*50)
1	c.4569_4573delinsT	p.(Ser1524Lysfs*23)
1	c.68_69del	p.(Glu23Valfs*17)
1	c.3331_3334del	p.(Gln1111Asnfs*5)
1	c.3481_3491del	p.(Glu1161Phefs*3)
1	c.3770_3771del	p.(Glu1257Glyfs*9)
1	c.798_799del	p.(Ser267Lysfs*19)
1	c.191G>A	p.(Cys64Tyr)
1	c.3481_3491del	p.(Glu1161Phefs*3)
1	c.[4281_4282insT; 4244_4281dup]	p.(Ser1428*)
1	c.134+3A>C	p.Cys27*
1	c.191G>A	p.(Cys64Tyr)
1	c.1480C>T	p.(Gln494*)
1	c.4016_4017insTT	p.(Glu1339Aspfs*28)
1	c.5123C>A	p.(Ala1708Glu)
1	c.5106delA	p.(Lys1702Asnfs*4)
1	c.5266dup	p.(Gln1756Profs*74)
1	c.4258C>T	p.(Gln1420*)
1	c.2217dup	p.(Val740Serfs*3)
1	c.4372C>T	p.(Gln1458*)
1	c.5080G>T	p.Asp1692_Trp1718delinsGly
1	c.5266dup	p.(Gln1756Profs*74)
1	c.5106del	p.(Lys1702Asnfs*4)
1	c.2603C>G	p.(Ser868*)
1	c.3756_3759del	p.(Ser1253Argfs*10)
1	c.4810C>T	p.(Gln1604*)
1	c.4391del	p.(Pro1464Leufs*2)
1	c.3331_3334del	p.(Gln1111Asnfs*5)
1	c.4391del	p.(Pro1464Leufs*2)
1	c.212+1G>A	p.Cys64*
1	c.3282T>G	p.(Tyr1094*)
1	c.3937C>T	p.(Gln1313*)

1	c.4569_4573delinsT	p.(Ser1524Lysfs*23)
1	c.3937C>T	p.(Gln1313*)
1	c.1115G>A	p.(Trp372*)
1	c.4065_4068del	p.(Asn1355Lysfs*10)
1	c.1961del	p.(Lys654Serfs*47)
1	c.2750del	p.(Ile917Thrfs*83)
1	c.3841C>T	p.(Gln1281*)
1	c.3770_3771del	p.(Glu1257Glyfs*9)
1	c.[4281_4282insT; 4244_4281dup]	p.(Ser1428*)
1	c.5194-2A>G	p.[His1732_Lys1759del,His1732Serfs*29]
1	c.1687C>T	p.(Gln563*)
1	c.5251C>T	p.(Arg1751*)
1	c.2524dup	p.(Glu842Glyfs*10)
1	c.2603C>G	p.(Ser868*)
1	c.1687C>T	p.(Gln563*)
1	c.5266dup	p.(Gln1756Profs*74)
1	c.4065_4068del	p.(Asn1355Lysfs*10)
1	c.(5332+?_5333-?)_(5406+?_5407-?)del	p.(Asp1778Glyfs*27)
1	c.5361_5362del	p.(Cys1787Trpfs*42)
1	c.3841C>T	p.(Gln1281*)
1	c.1979_1982del	p.(Val660Glyfs*40)
1	c.181T>G	p.(Cys61Gly
1	c.(441+?_442-?)_(4357+?_4358-?)del	p.(Glu149Tyrfs*2)
1	c.5030_5033del	p.(Thr1677Ilefs*2)
1	c.815_824dup	p.(Thr276Alafs*14)
1	c.5266dup	p.(Gln1756Profs*74)
1	c.(441+?_442-?)_(4357+?_4358-?)del	p.(Glu149Tyrfs*2)
1	c.4391delC	p.(Pro1464Leufs*2)
1	c.3937C>T	p.(Gln1313*)
1	c.3481_3491del	p.(Glu1161Phefs*3)
1	c.3770_3771del	p.(Glu1257Glyfs*9)
1	c.3481_3491del	p.(Glu1161Phefs*3)
1	c.134+3A>C	p.Cys27*
1	c.191G>A	p.(Cys64Tyr)
1	c.4183C>T	p.Gln1395*
1	c.3481_3491del	p.(Glu1161Phefs*3)
1	c.843_846del	p.(Ser282Tyrfs*15)
1	c.3770_3771del	p.(Glu1257Glyfs*9)
1	c.5266dup	p.(Gln1756Profs*74)
1	c.3839_3843delinsAGGC	p.(Ser1280*)
1	c.5266dup	p.(Gln1756Profs*74)
1	c.843_846del	p.(Ser282Tyrfs*15)
1	c.1292T>G	p.(Leu431*)
1	c.3756_3759del	p.(Ser1253Argfs*10)
1	c.5266dup	p.(Gln1756Profs*74)

1	c.3839_3843delinsAGGC	p.(Ser1280*)
1	c.212+3A>G	p.Cys64*
1	c.3756_3759del	p.(Ser1253Argfs*10)
1	c.5074+1G>A	p.[Asp1692Glyfs*14,Val1665Serfs*8]
1	c.2092del	p.(Leu698Tyrfs*32)
1	c.181T>G	p.Cyst61Gly
1	c.4065_4068del	p.(Asn1355Lysfs*10)
1	c.1961del	p.(Lys654Serfs*47)
1	c.68_69del	p.(Glu23Valfs*17)
1	c.5537_5556del	p.(Gln1846Leufs*27)
1	c.3756_3759del	p.(Ser1253Argfs*10)
1	c.4065_4068del	p.(Asn1355Lysfs*10)
1	c.(4484+1_4485-1)_(4675+1_4676-1)del	p.(Ser1496Glyfs*14)
1	c.3381T>G	p.(Tyr1127*)
1	c.1121del	p.(Thr374Asnfs*2)
1	c.4222C>T	p.Gln1408*
1	c.19_47del	p.(Arg7Cysfs*24)
1	c.815_824dup	p.(Thr276Alafs*14)
1	c.2066_2069del	p.(Ser689Lysfs*11)
1	c.2612delinsTT	p.(Pro871Leufs*32)
1	c.1729_1730del	p.(Glu577Ilefs*8)
1	c.213-2A>C	p.Arg71Serfs*10
1	c.2138C>G	p.(Ser713*)
1	c.212+3A>G	p.Cys64*
1	c.5123C>A	p.(Ala1708Glu)
1	c.5035_5039del	p.(Leu1679Tyrfs*2)
1	c.3756_3759del	p.(Ser1253Argfs*10)
1	c.1121del	p.(Thr374Asnfs*2)
1	c.4216A>T	p.(Lys1406*)
1	c.1A>G	p.(Met1?)
1	c.4675G>A	p.Gln1556Glyfs*14
1	c.5296del	p.(Ile1766Serfs*27)
1	c.5080G>T	p.Asp1692_Trp1718delinsGly
1	c.1965C>A	p.(Tyr655*)
1	c.4183C>T	p.(Gln1395*)
1	c.140G>T	p.(Cys47Phe)
1	c.5537_5556del	p.(Gln1846Leufs*27)
1	c.5266dup	p.(Gln1756Profs*74)
1	c.3700_3704del	p.(Val1234Glnfs*8)
1	c.5177_5180del	p.(Arg1726Lysfs*3)
1	c.5123C>A	p.(Ala1708Glu)
1	c.34C>T	p.(Gln12*)
1	c.68_69del	p.(Glu23Valfs*17)
1	c.3756_3759del	p.(Ser1253Argfs*10)
1	c.1961del	p.(Lys654Serfs*47)

1	c.2217dup	p.(Val740Serfs*3)
1	c.5266dup	p.(Gln1756Profs*74)
1	c.(5074+1_5075-1)_(5277+1_5278-1)dup	p.?
1	c.220C>T	p.(Gln74*)
1	c.5289del	p.(Leu1764*)
1	c.5266dup	p.(Gln1756Profs*74)
1	c.5072C>A	p.(Thr1691Lys)
1	c.5213 G>T	p.(Gly1738Val)
1	c.4161_4162del	p.(Gln1388Glufs*2)
1	c.1513A>T	p.(Lys505*)
1	c.140G>T	p.(Cys47Phe)
1	c.3013del	p.(Glu1005Asnfs*19)
1	c.1505_1508del	p.(Leu502Cysfs*29)
1	c.(4185+1_4186-1)_(4357+1_4358-1)dup	p.?
1	c.5266dup	p.(Gln1756Profs*74)
1	c.188T>A	p.(Leu63*)
1	c.5266dup	p.(Gln1756Profs*74)
1	c.211A>G	p.[Cys64*;Phe46_Arg71Gly]
1	c.5080G>T	p.Asp1692_Trp1718delinsGly
1	c.68_69del	p.(Glu23Valfs*17)
1	c.5089T>C	p.(Cys1697Arg)
1	c.3481_3491del	p.(Glu1161Phefs*3)
1	c.2612_2613insT	p.(Phe872Valfs*31)
1	c.3481_3491del	p.(Glu1161Phefs*3)
1	c.66dup	p.(Glu23Argfs*18)
1	c.5080G>T	p.Asp1692_Trp1718delinsGly
1	c.4327C>T	p.(Arg1443*)
1	c.(5332+1_5333-1_5406+1_5407-1)del	p.?
1	c.815_824dup	p.(Thr276Alafs*14)
1	c.5266dup	p.(Gln1756Profs*74)
1	c.5080G>T	p.Asp1692_Trp1718delinsGly
1	c.68_69del	p.(Glu23Valfs*17)
1	c.2679_2682del	p.(Lys893Asnfs*106)
1	c.2068A>T	p.(Lys690*)
1	c.(80+1_81-1)_(441+1_442-1)dup	p.?
1	c.4185G>A	p.Gly1366Alafs*8
1	c.3841C>T	p.(Gln1281*)
1	c.367del	p.(Ser123Leufs*40)
1	c.68_69del	p.(Glu23Valfs*17)
1	c.5419del	p.(Ile1807Leufs*27)
2	c.145G>T	p.(Glu49*)
2	c.8639_8640del	p.(Thr2880Asnfs*26)
2	c.8364G>A	p.(Trp2788*)
2	c.6079dup	p.(Arg2027Lysfs*22)
2	c.8485C>T	p.(Gln2829*)

2	c.8219T>G	p.(Leu2740*)
2	c.6447_6448dup	p.(Lys2150Ilefs*19)
2	c.658_659del	p.(Val220Ilefs*4)
2	c.771_775del	p.(Asn257Lysfs*17)
2	c.7680dup	p.(Gln2561Serfs*5)
2	c.1813dupA	p.(Ile605Asnfs*11)
2	c.3303_3336dup	p.(Glu1113*)
2	c.1929del	p.(Arg645Glufs*15)
2	c.1981_1984dup	p.(Ser662*)
2	c.6591_6592del	p.(Glu2198Asnfs*4)
2	c.9253dup	p.(Thr3085Asnfs*26)
2	c.1813dup	p.(Ile605Asnfs*11)
2	c.(8331+1_8332-1)_(8632+1_8633-1)del	p.?
2	c.5984dup	p.(Asn1995Lysfs*8)
2	c.4037_4038del	p.(Thr1346Serfs*5)
2	c.6626_6627del	p.(Ile2209Argfs*15)
2	c.8364G>A	p.(Trp2788*)
2	c.9672dup	p.(Tyr3225Ilefs*30)
2	c.2133C>A	p.(Cys711*)
2	c.5217_5220del	p.(Tyr1739*)
2	c.6509_6510del	p.(Lys2170Serfs*5)
2	c.6408_6414del	p.(Asn2137Lysfs*29)
2	c.272delinsTT	p.(Tyr91Phefs*10)
2	c.9403del	p.(Leu3135Phefs*28)
2	c.3847_3848del	p.(Val1283Lysfs*2)
2	c.9294C>A	p.(Tyr3098*)
2	c.9097del	p.(Thr3033Leufs*29)
2	c.[3637delG;3641T>G]	p.(Glu1213Lysfs*15)
2	c.9294C>A	p.(Tyr3098*)
2	c.2092del	p.(Leu698Tyrfs*32)
2	c.771_775del	p.(Asn257Lysfs*17)
2	c.7654dup	p.(Ile2552Asnfs*2)
2	c.9501+3A>T	p.Gly3086Glufs*3
2	c.5722_5723del	p.(Leu1908Argfs*2)
2	c.5946del	p.(Ser1982Argfs*22)
2	c.1310_1313del	p.(Lys437Ilefs*22)
2	c.755_758del	p.(Asp252Valfs*24)
2	c.2701del	p.(Ala902Leufs*2)
2	c.5609_5610delinsAG	p.(Phe1870*)
2	c.2548C>T	p.(Gln850*)
2	c.3195_3198del	p.(Asn1066Leufs*10)
2	c.2612C>A	p.(Ser871*)
2	c.6626_6627delTA	p.(Ile2209Argfs*15)
2	c.5157_5161del	p.(Asn1719Lysfs*6)
2	c.3847_3848del	p.(Val1283Lysfs*2)

2	c.9061G>T	p.(Glu3021*)
2	c.5946del	p.(Ser1982Argfs*22)
2	c.5566_5584del	p.(His1856*)
2	c.9097del	p.(Thr3033Leufs*29)
2	c.9294C>A	p.(Tyr3098*)
2	c.9294C>A	p.(Tyr3098*)
2	c.8537_8538del	p.(Glu2846Glyfs*22)
2	c.658_659del	p.(Val220Ilefs*4)
2	c.1310_1313del	p.(Lys437Ilefs*22)
2	c.771_775del	p.(Asn257Lysfs*17)
2	c.1813dup	p.(Ile605Asnfs*11)
2	c.1212del	p.(Asn404Lysfs*26)
2	c.736_755del	p.(Phe246Glnfs*2)
2	c.6833_6837del	p.(Ile2278Serfs*13)
2	c.9294C>A	p.(Tyr3098*)
2	c.7007G>C	p.Gly2281Alafs*31
2	c.7436-2A>T	p.Asp2479Valfs*41
2	c.5482_5486del	p.(Lys1828Valfs*4)
2	c.(67+1_68-1)_(316+1_317-1)del	p.?
2	c.5645C>A	p.(Ser1882*)
2	c.5439dup	p.(Val1814Cysfs*3)
2	c.1832C>A	p.(Ser611*)
2	c.5645C>A	p.(Ser1882*)
2	c.6841+1del	p.(Gly2281Glufs*10)
2	c.6405_6409del	p.(Asn2135Lysfs*3)
2	c.3386dup	p.(Phe1130Valfs*2)
2	c.4889C>G	p.(Ser1630*)
2	c.8995_8996del	p.(Leu2999Valfs*18)
2	c.5645C>A	p.(Ser1882*)
2	c.9398C>G	p.(Ser3133*)
2	c.9274del	p.(Tyr3092Ilefs*12)
2	c.3847_3848del	p.(Val1283Lysfs*2)
2	c.5653dup	p.(Cys1885Leufs*15)
2	c.4648G>T	p.(Glu1550*)
2	c.7506dup	p.(Val2503Argfs*36)
2	c.3847_3848del	p.(Val1283Lysfs*2)
2	c.3812C>G	p.(Ser1271*)
2	c.7234_7235insG	p.(Thr2412Serfs*2)
2	c.203_218del	p.(Lys68Serfs*7)
2	c.2T>G	p.(Met1?)
2	c.6444dup	p.(Ile2149Tyrfs*2)
2	c.5645C>A	p.(Ser1882*)
2	c.2808_2811del	p.(Ala938Profs*21)
2	c.5645C>A	p.(Ser1882*)

Supplementary Table S2: Patients characteristics in the triple-negative breast cancers (TNBC) cohort. *NA*: not-available. *** Positive nodes if pre-chemotherapy biopsy positive or there was at least one yN or scar in the removed node after neoadjuvant chemotherapy.

**Variable**	**All (n=270)**	***BRCA* status**			***p***
		**Non-carriers (n = 148)**	***BRCA1* (n=106)**	***BRCA2* (n = 16)**	
**Age, *years*, median (25th-75th) *NA=0***	41 (35-49)	41 (35-49)	39 (34-47)	45 (49-51)	0.066
**cT *(%)*<br>cT0<br>cT1<br>cT2<br>cT3<br>cT4<br>*NA=63***	7 (3%)<br>69 (33%)<br>93 (45%)<br>30 (14%)<br>8 (4%)	2 (2%)<br>31 (29%)<br>51 (46%)<br>19 (17%)<br>6(5%)	4 (5%)<br>33 (38%)<br>38 (44%)<br>9 (10%)<br>2 (2%)	1 (9%)<br>4 (36%)<br>4 (36%)<br>2 (18%)<br>0 (0%)	0.52
**cN *(%)*<br>cN0<br>cN1<br>cN2<br>cN3<br>*NA=67***	145 (71%)<br>53 (26%)<br>3 (1%)<br>2 (1%)	70 (65%)<br>34 (31%)<br>2 (2%)<br>2 (2%)	67 (79%)<br>17 (20%)<br>1 (1%)<br>0 (0%)	8 (80%)<br>2 (20%)<br>0 (0%)<br>0 (0%)	0.42
**Positive nodes ***<br>NA= 11**	73 (28%)	44 (31%)	26 (26%)	3 (19%)	0.57
**Grade *(%)*<br>1<br>2<br>3<br>*NA=3***	3 (1%)<br>44 (16%)<br>220 (82%)	2 (1%)<br>29 (20%)<br>116 (79%)	1 (1%)<br>12 (12%)<br>80 (88%)	0 (0%)<br>3 (19%)<br>13 (81%)	0.50
**Mitotic index *(%)*<br>1<br>2<br>3<br>*NA=24***	25 (10%)<br>52 (21%)<br>169 (69%)	20 (14%)<br>27 (19%)<br>93 (66%)	4 (4%)<br>24 (26%)<br>66 (70%)	1 (8%)<br>1 (8%)<br>10 (83%)	0.087
**Chemotherapy *(%)*<br>Neoadjuvant<br>Adjuvant<br>Both<br>*NA=0***	89 (33%)<br>179 (66%)<br>2 (1%)	52 (35%)<br>95 (64%)<br>1 (1%)	30 (28%)<br>75 (71%)<br>1 (1%)	7 (44%)<br>9 (56%)<br>0 (0%)	0.54
**Anthracyclines *(%)* *NA=2***	237 (88%)	129 (88%)	96 (91%)	12 (75%)	0.13
**Taxanes *(%) NA=2***	220 (82%)	130 (88%)	78 (74%)	12 (75%)	0.0088
**Alkylating agent *(%)* *NA=2***	264 (99%)	145 (99%)	103 (98%)	16 (100%)	1
**Platinum *(%) NA=2***	3 (1%)	0 (0%)	2 (2%)	1 (6%)	0.037

Supplementary Table S3: Patients characteristics in the non-TNBC cohort.

Variable	All (n=588)	*BRCA* status			*p*
		Non-carriers (n = 472)	*BRCA1* (n=49)	*BRCA2* (n = 67)	
**Age, years, median (25th-75th)** ***NA=0***	39 (34-47)	39 (34-47)	45 (37-50)	39 (34-46)	0.014
**cT** ***(%)***					0.58
**cT0**	36 (8%)	32 (9%)	2 (6%)	2 (4%)	
**cT1**	167 (37%)	131 (36%)	19 (49%)	17 (33%)	
**cT2**	181 (40%)	147 (40%)	12 (31%)	22 (43%)	
**cT3**	53 (12%)	43 (12%)	4 (10%)	6 (12%)	
**cT4**	19 (4%)	13 (4%)	2 (5%)	4 (8%)	
NA=133					
**cN** ***(%)***					0.59
**cN0**	298 (66%)	237 (65%)	28 (72%)	33 (65%)	
**cN1**	145 (32%)	119 (33%)	11 (28%)	15 (29%)	
**cN2**	5 (1%)	4 (1%)	0 (0%)	1 (2%)	
**cN3**	6 (1%)	4 (1%)	0 (0%)	2 (4%)	
NA=135					
**Positive nodes***** ***NA=*** **10**	322 (56%)	251 (54%)	29 (60%)	42 (64%)	0.28
**Grade** ***(%)***					0.056
**1**	37 (6%)	34 (7%)	0 (0%)	3 (5%)	
**2**	275 (48%)	226 (49%)	17 (36%)	32 (51%)	
**3**	262 (46%)	204 (44%)	30 (64%)	28 (44%)	
NA=14					
**Mitotic index** ***(%)***					0.047
**1**	183 (35%)	155 (36%)	7 (17%)	21 (36%)	
**2**	179 (34%)	145 (34%)	14 (33%)	20 (34%)	
**3**	164 (31%)	125 (29%)	21 (50%)	17 (29%)	
NA=63					
**Positive ER** ***(%) NA=0***	516 (88%)	417 (88%)	36 (73%)	63 (94%)	0.0053
**Positive PR** ***(%) NA=0***	449 (76%)	360 (76%)	34 (69%)	55 (82%)	0.29
**Positive HER-2** ***(%) NA=0***	173 (29%)	153 (32%)	7 (14%)	13 (19%)	0.0040
**Chemotherapy** ***(%)***					0.60
**Neoadjuvant**	154 (26%)	125 (26%)	9 (18%)	20 (30%)	
**Adjuvant**	428 (73%)	341 (72%)	40 (82%)	47 (70%)	
**Both**	6 (1%)	6 (1%)	0 (0%)	0 (0%)	
NA=0					
**Anthracyclines** ***(%) NA=1***	457 (78%)	368 (78%)	33 (67%)	56 (85%)	0.083
**Taxanes** ***(%) NA=1***	483 (82%)	389 (82%)	39 (80%)	55 (83%)	0.82
**Alkylating agent** ***(%) NA=2***	547 (93%)	439 (93%)	44 (90%)	64 (97%)	0.29
**Platinum** ***(%) NA=1***	27 (5%)	23 (5%)	3 (6%)	1 (1%)	0.35
**Trastuzumab** ***(%) NA=1***	142 (24%)	130 (28%)	3 (6%)	9 (14%)	0.00014

Supplementary Table S4: Landmark analysis at one year of the impact of *BRCA* status on DFS and DSS in the TNBC cohort

**Cox proportional hazards regression for disease-free-survival**			
		**Unadjusted analysis**	
	**N**	**HR (95%CI)**	***p***
**Non-carriers**	148 (55%)	1	0.010
BRCA1	106 (39%)	0.48 (0.28-0.83)	
BRCA2	16 (6%)	0.34 (0.10-1.16)	
**Cox proportional hazards regression for disease-specific survival**			
		**Unadjusted analysis**	
	**N**	**HR (95%CI)**	***p***
**Non-carriers**	148 (55%)	1	0.032
BRCA1	106 (39%)	0.46 (0.24-0.87)	
BRCA2	16 (6%)	0.40 (0.10-1.7)	

Supplementary Table S5: Multivariate analysis of DFS and DSS in the subgroup of patients with non-TNBC. NI: not included

Cox proportional hazards regression											
		Disease-free survival					Disease-specific survival				
			Unadjusted analysis		Adjusted Analysis			Unadjusted analysis		Adjusted Analysis	
	**N**	**5-years DFS rate (95% CI)**	**HR (95%CI)**	***p***	**HR (95%CI)**	***p***	**5-years DSS rate (95% CI)**	**HR (95%CI)**	***p***	**HR (95%CI)**	***p***
**Non-carriers**	472 (81%)	92 (90-95)	1				97 (95-99)	1		1	
BRCA1	49 (8%)	91 (82-100)	0.91 (0.50-1.7)	0.88	NI	NI	97 (92-100)	0.86 (0.37-2.0)	0.93	0.90 (0.39-2.1)	0.89
BRCA2	67 (11%)	87 (78-96)	1.1 (0.70-1.9)				98 (95-100)	1.0 (0.51-2.1)		0.97 (0.48-2.0)	
**Grade**											
**1**	37 (6%)	97 (92-100)	1				94 (92-96)	1	0.68	NI	NI
**2**	275 (48%)	92 (89-96)	1.4 (0.70-2.8)	0.60	NI	NI	93 (86-100)	1.5 (0.53-4.2)			
**3**	262 (46%)	90 (86-94)	1.4 (0.68-2.8)				91 (85-99)	1.4 (0.48-3.8)			
**Age**											
**>35**	413 (70%)	93 (90-96)	1	0.086	NI	NI	95 (92-97)	1	0.030	1	0.032
**≤35**	175 (30%)	88 (83-94)	1.4 (1.0-1.9)				91 (87-96)	1.7 (1.1-2.6)		1.7 (1.0-2.6)	
**Nodal status**											
**Negative**	256 (44%)	94 (90-97)	1	0.082	NI	NI	96 (94-99)	1	0.0018	1	0.0015
**Positive**	322 (56%)	90 (86-93)	1.4 (0.96-1.9)				92 (89-95)	2.3 (1.3-3.8)		2.3 (1.4-3.9)	

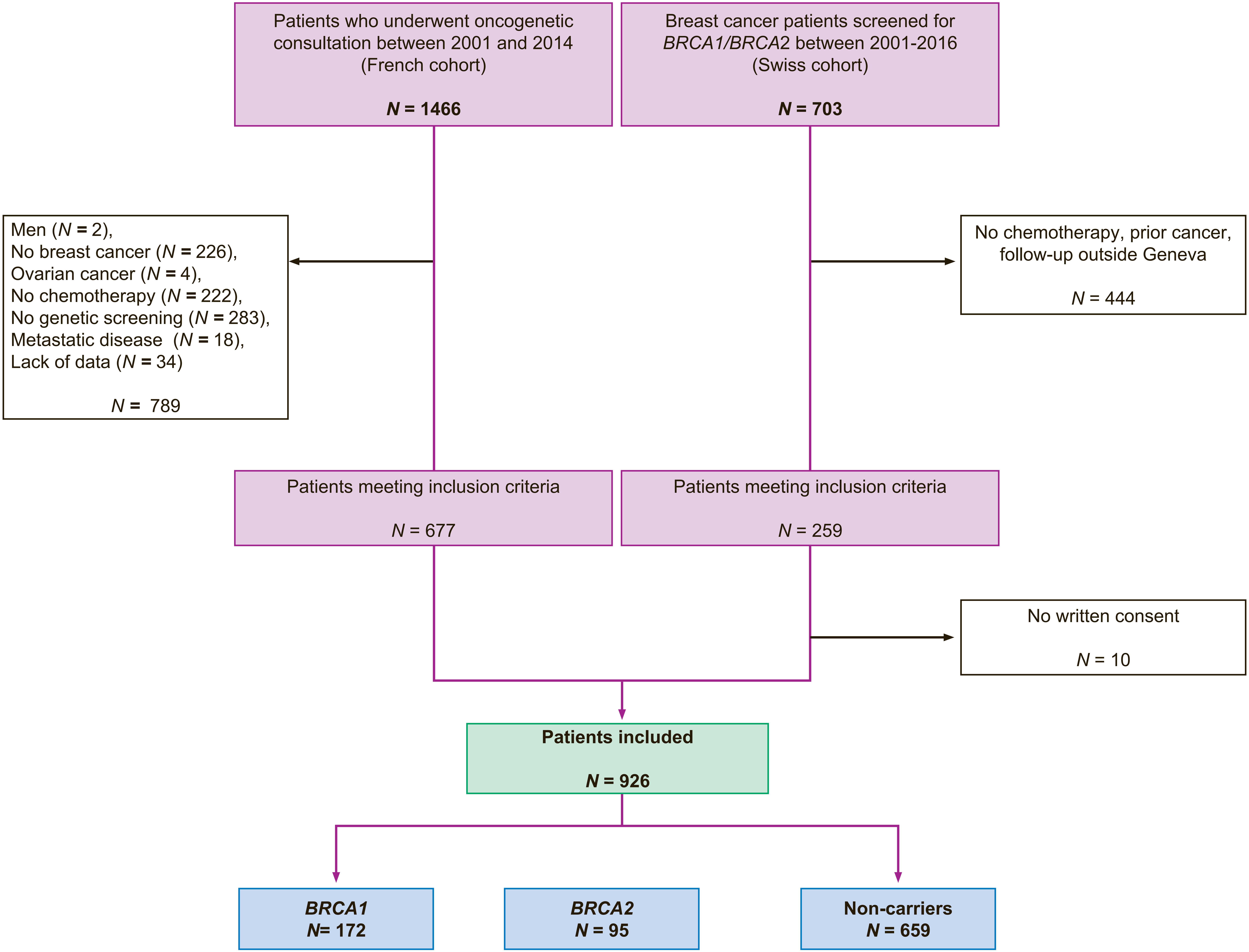
Patients who underwent oncogenetic consultation between 2001 and 2014 (French cohort)
N = 1466
Breast cancer patients screened for BRCA1/BRCA2 between 2001-2016 (Swiss cohort)
N = 703
Men (N = 2),
No breast cancer (N = 226),
Ovarian cancer (N = 4),
No chemotherapy (N = 222),
No genetic screening (N = 283),
Metastatic disease (N = 18),
Lack of data (N = 34)
N = 789
No chemotherapy, prior cancer, follow-up outside Geneva
N = 444
Patients meeting inclusion criteria
N = 677
Patients meeting inclusion criteria
N = 259
No written consent
N = 10
Patients included
N = 926
BRCA1
N= 172
BRCA2
N = 95
Non-carriers
N = 659
